# National Needs Assessment of Emergency Medicine Residencies for Musculoskeletal Knowledge

**DOI:** 10.7759/cureus.43638

**Published:** 2023-08-17

**Authors:** William Denq, Alexander J Tomesch, Allison D Lane, Aaron Thomas, Neil L McNinch, Anna Waterbrook

**Affiliations:** 1 Emergency Medicine, University of Arizona College of Medicine - Tucson, Tucson, USA; 2 Emergency Medicine/Sports Medicine, University of Missouri, Columbia, USA; 3 Emergency Medicine/Sports Medicine, University of Arizona College of Medicine - Tucson, Tucson, USA; 4 Emergency Medicine/Sports Medicine, Mayo Clinic, Phoenix, USA; 5 Statistics, McNinch Biostats, LLC, Kent, USA

**Keywords:** musculoskeletal (msk) complaints and injuries, american board of emergency medicine, electronic survey, orthopedics, sports medicine rotation, emergency medicine resident, knowledge assessment, musculoskeletal education

## Abstract

Introduction

Musculoskeletal (MSK) complaints and injuries account for a large percentage of presenting chief complaints to the emergency department in the United States (US). Despite the prevalence and economic impact on the US healthcare system, there is a documented deficiency in MSK education at all training and practicing levels in the US medical system. The purpose of this needs assessment is to better determine the state of MSK education in Emergency Medicine residency programs.

Methods

An online needs assessment form was sent to Emergency Medicine program directors in the US. Summary statistics were performed followed by an exploratory analysis.

Results

Data from 43 of 272 Emergency Medicine program directors that responded to this needs assessment were analyzed. Respondents ranked the importance of MSK education in Emergency Medicine on a Likert scale of 1-5 (very unimportant to very important) at a mean of 4.2. Additionally, 97.6% of respondents believe that their MSK curriculum could be improved. Seventy-nine percent of respondents were somewhat likely or highly likely to use a standardized method or tool to assess MSK knowledge. Of the top three barriers to MSK education implementation, 94.9% cited time, 56.4% cited interdepartmental relations, and 46.2% cited funding.

Conclusion

MSK knowledge is taught and assessed in highly variable methods across Emergency Medicine residency programs. Although efforts are being made to address the known deficiency in MSK knowledge, further research is needed to perform a larger needs assessment, study innovative MSK education modalities, and develop a standardized MSK assessment for Emergency Medicine residency training.

## Introduction

Musculoskeletal (MSK) complaints and injuries are one of the most common reasons that patients present to United States (US) emergency departments (ED) for care [1,2]. Despite the high prevalence and economic burden of these disorders, previous studies describe a deficiency in MSK education at all training levels and among practicing physicians in the US medical system [3-10]. Emergency physicians (EPs) are no exception. Previous studies have documented low passing rates on a validated MSK examination (Freedman and Bernstein (FB)-MSK) and reported emergency medicine (EM) residency graduates not feeling well prepared to care for MSK complaints [9,11]. 

There is a growing body of literature that attempts to address this educational deficiency among EPs. This includes descriptions of the integration of primary care sports medicine (PCSM) rotation for EM residents into their residency curriculums, as well as MSK knowledge acquisition before and after both PCSM and traditional orthopedic rotations using the FB-MSK [12-14]. The American Society for Sports Medicine (AMSSM) also recently published recommended curricular guidelines for MSK and sports medicine (SM) in EM residency [15]. In addition, the EM Council of Residency Directors (CORD) has published an online SM toolkit offering modules, videos, and case presentations as a “plug and play” SM curriculum for EM residency programs [16].

While there is increased recognition of the need for better MSK EP education as well as the ability to address it, there has never been a formal needs assessment of MSK knowledge, training, and educational barriers in current EM residency programs. This information will be vital as we begin to expand MSK curricula and programs for EP education.

## Materials and methods

An electronic needs assessment survey was created to collect data about MSK education in EM residency training programs. Subject matter experts comprising three EM/SM attending physicians, one EM/SM fellow, and one family medicine/SM fellow at the University of Arizona, Tucson, Arizona, United States, developed and reviewed questions pertinent to this assessment. Formatting of the needs assessment was founded in step 1 of Kern’s six-step approach to curriculum development: problem identification and general needs assessment [17].

The identified problem was labeled as MSK education deficiency among EM residents. The general needs assessment format from Kern asks what is currently being done, what personal and environmental factors affect the problem, what ideally should be done, and what are the key differences between current and ideal. The developed assessment questions mirrored these by inquiring about current strategies for MSK education, perceived barriers to MSK education, program needs regarding MSK education, and what should be done to improve MSK education.

Program demographic questions included the presence of variables such as an MSK rotation, orthopedic residency, or EM/SM faculty to determine if they had any influence on perceived barriers or needs. MSK knowledge domains of disease treatment, imaging, pathology, examination, and splinting were agreed upon by subject matter experts as generalizable areas of education. Educational formats, locations, and assessment methods were similarly developed. However, the option to provide other answers was given with each question in the event there was an unexpected response.

The assessment was distributed electronically from November 2021 to January 2022 via Qualtrics (Seattle, Washington, United States) to EM program directors throughout the US, derived from the CORD listserv. A reminder regarding the assessment was sent approximately one month after the initial request. A total of 43 respondents participated with a 15.8% response rate. These anonymous responses were collected, and the results were analyzed.

The survey analysis was mainly descriptive in nature. The examination of data began with the calculation of summary statistics: frequencies and percentages for categorical data, and median (interquartile range (IQR)) for non-normally distributed continuous and rank data. Exploratory analyses used the Wilcoxon Rank Sum Test, Kruskal-Wallis Test, and Chi-Square Test of Independence to examine differences and associations by response. Analyses were conducted using Stata Statistical Software: Release 17 (2021; StataCorp LLC, College Station, Texas, US) [18].

This project was reviewed by the University of Arizona Institutional Review Board and approved as an exemption (STUDY00000261, dated December 17, 2021).

## Results

The demographic data of the respondents can be found in Table 1.

**Table 1:** Demographic data of the respondents MSK: musculoskeletal; EM: emergency medicine; SM: sports medicine; IQR: interquartile range

	n	Median or Percentage	IQR
Program Location			
Midwest	10	23.2%	-
Northeast	9	20.9%	-
Southeast	14	32.6%	-
Southwest	5	11.6%	-
West/Northwest	5	11.6%	-
Program Makeup			
Academic	43	60%	20-90
Community	43	20%	5-72.5
Annual Emergency Department volume	41	75,000	60,000-90,000
Residency length (years)			
Three	39	86.7%	-
Four	6	13.3%	-
Number of hospital rotations	43	3	2-4
Presence of MSK rotation	43		
Yes	30	69.8%	-
No	13	30.2%	-
Presence of Orthopedic Surgery residency	43	69.8%	
Yes	30	69.8%	-
No	13	30.2%	-
Presence of Primary Care Sports Medicine fellowship	43		
Yes	17	39.5%	-
No	26	60.5%	-
Presence of EM/SM faculty	43		
Yes	17	39.5%	-
No	26	60.5%	-
Number of EM/SM faculty	10	1	1-3.25

On average, Orthopedic Surgery manages 49.8% of fracture care in the ED, EM manages 48.8% of fracture care, and Podiatry or Plastic Surgery manages 1.4% of fracture care. Regarding MSK education, 100% of respondents’ residencies teach MSK topics and it averages 10.4% (5-20%) of their entire curriculum. See Table 2 for an overview of the respondents’ MSK curriculum.

**Table 2:** MSK Curriculum and Assessment Response MSK: musculoskeletal

MSK Curriculum	n (%)	Response
MSK Knowledge Domains	41 (95.3)	MSK disease treatment
	43 (100)	MSK imaging
	38 (88.4)	MSK pathology
	39 (90.7)	MSK examination
	42 (97.7)	Splinting
Educational Formats	29 (67.4)	Required MSK rotation
	25 (86.2)	Orthopedics
	4 (13.8)	Sports Medicine
	11 (25.6)	Elective MSK rotation
	3 (11.1)	Orthopedics
	24 (88.9)	Sports Medicine
Location of required MSK rotations	20 (69.0)	Emergency Department
	16 (55.2)	Orthopedics inpatient
	10 (34.5)	Orthopedics outpatient
	12 (41.4)	Sports Medicine outpatient
	1 (3.5)	Ski Patrol
	1 (3.5)	Ortho Tech and Fracture Conference
Assessment Methods	37 (86.0)	Faculty Assessment
	39 (90.7)	In-Service Evaluation
	22 (51.2)	Oral Cases
	8 (18.6)	Written Exam
	1 (2.3)	Splint Lab
	2 (4.7)	Online Question Bank
Utilization of specific MSK assessment	2 (4.7)	Yes
	41 (95.3)	No

Respondents ranked the importance of MSK education in EM on a Likert scale of 1-5, very unimportant to very important, at a mean of 4.2. Regarding their MSK curriculum, 97.6% of respondents believed that it could be improved. The two respondents that utilized a specific MSK assessment cited using Rosh Review and Physician's Evaluation and Educational Review in Emergency Medicine (PEER). Seventy-nine percent of respondents were somewhat likely or highly likely to use a standardized method or tool to assess MSK knowledge. See Table 3 for identified deficits and barriers.

**Table 3:** Identified deficits and barriers to MSK education MSK: musculoskeletal

	n (%)	Responses
MSK Domains Needing Improvement	29 (70.7)	MSK disease treatment
	27 (65.9)	MSK imaging
	18 (43.9)	MSK pathology
	28 (68.3)	MSK examination
	18 (43.9)	Splinting
	4 (9.8)	Reductions
Educational Formats Wanted	10 (25.0)	Required MSK rotation
	13 (32.5)	Elective MSK rotation
	17 (42.5)	Lecture
	25 (62.5)	On-shift teaching
	32 (80)	Simulation
	33 (82.5)	Workshop
Barriers to Implementation	18 (46.2)	Funding
	22 (56.4)	Interdepartmental relations
	12 (30.8)	Lack of expertise
	37 (94.9)	Time
	1 (2.6)	Quality of rotation
	1 (2.6)	EM faculty comfort

Six of seven comments from the survey cited orthopedic surgery involvement in the ED as a barrier. Respondents cited orthopedic consult culture as limiting MSK experiences for EM residents, especially in academic centers. Either a heavily involved orthopedic consult service or EP comfortable with orthopedic management were described as contributing to this limitation. One comment cited the loss of EM/SM involvement as a barrier.

A Chi-Square Test of Independence determined the following results: the barrier of time is not significantly dependent on the number of years of residency (p=0.164), the barrier of lack of expertise was not significantly dependent on the presence of EM/SM faculty (p=0.542), the barrier of lack of expertise or interdepartmental relations was not significantly dependent on the presence of an MSK rotation (p=0.087), and the percentage of MSK curriculum is significantly dependent on the presence of SM faculty (p=0.033).

The Kruskal-Wallis Test was used to assess differences based on the presence of interdepartmental relations as a barrier impeding the implementation of improvements in MSK education. There were significant differences found between this barrier and percentage fracture management when performed by orthopedics (p-value = 0.005) versus emergency medicine (p= 0.001).

## Discussion

This needs assessment among EM program directors demonstrates an existing difficulty in assessing and teaching MSK knowledge for a variety of reasons. MSK education is perceived to be important by respondents. Simultaneously, the vast majority of programs believe their MSK curriculum can be improved. Domains of emergency MSK education such as MSK disease treatment, examination, and imaging were among the top cited areas needing improvement. 

Although disease treatment can be taught through multiple educational modalities, MSK examination and imaging may require significant investment in time and volume. This can stem from a lack of foundational knowledge starting in medical school where there is a commonly reported deficit in MSK education [5,8,19,20]. This deficit was initially identified in the late 1990s by Freedman et al. [9]. However, despite the acknowledgment of this deficit and continued research into this problem, MSK education is still being reported as a deficiency at the medical school level [5]. This inadequacy is not limited to the US training system; it is cited as an urgent need to address on the international level [21-23]. One study demonstrated a significant increase in MSK knowledge in fourth-year medical students after participation in a two-week clinical elective [20]. However, clinical confidence did not correlate with this increase in MSK knowledge. There can be several possibilities for this reason, but we conjecture that MSK clinical confidence is derived from all facets of MSK education. MSK physical examination techniques, splinting, and reductions are all skills that are difficult to assess from a standardized examination and may not be adequately covered in a two-week elective. More studies are needed to address this discrepancy.

EM residency programs have certainly recognized the need for additional MSK education beyond current medical school efforts. From this assessment, we learned most programs have a required MSK rotation and heavily utilize workshops, simulation, and on-shift teaching to further provide MSK educational opportunities. Despite being listed as a potential institutional barrier, collaboration with orthopedic colleagues represents a potential avenue for MSK education [24]. The lack of a standardized curriculum makes it difficult to consistently teach MSK knowledge throughout the country. In its current state, programs use a multitude of rotation types, locations, and lengths. Program directors indicated in the assessment that they would welcome improvements to MSK education. The AMSSM guidelines of 2021 recommend a variety of educational methods that range from hands-on teaching to online modules [15]. This correlates with the desired educational formats indicated by respondents. However, as cited by this assessment, several barriers prevent proper implementation: time, funding, interdepartmental relations, and lack of expertise. Beyond increasing residency length and funding, innovative methods to address these barriers are needed.

Two respondents reported using MSK rotations such as ski patrol or orthopedic tech and fracture conference to help. These educational methods, while unconventional and region-specific, could represent different methods that meet the needs of programs. Interestingly, programs reported wanting more hands-on education through simulation and workshops. The US Department of Veterans Affairs developed a pilot professional development program for primary care attending physicians, intending to improve the evaluation and management of patients with common MSK conditions [25]. This two-week MSK mini-residency used a multifaceted approach including hands-on teaching that resulted in evidence of cost-effectiveness, participant satisfaction, and an increase in joint injections following the course. Perhaps we can draw a parallel to the EM specialty and use aspects of this national course to create a standard for training EM residents that meets the needs of programs while addressing the barrier of time.

The only recognized EM standardized assessment tool is the in-training examination offered by the American Board of Emergency Medicine. Nontraumatic MSK knowledge is tested in 3% of the exam while a portion of the 9% in Traumatic Disorders tests traumatic MSK knowledge. Programs reported utilizing EM question banks to assess MSK knowledge. Two validated exams are targeted toward MSK knowledge. The FB-MSK was validated for medical school students by orthopedic chairs while the MSK-30 was validated by orthopedic surgeons, PCSM physicians, a family physician, a physical therapist, and PCSM fellows [9,26]. The creation of a standardized MSK knowledge exam specific to EM would be beneficial in helping assess emergency MSK knowledge in the specialty. Many of the respondents indicated they would use an assessment tool if one were created and available.

There are several limitations to this study. Despite our best efforts, the study was a primarily subjective assessment vulnerable to biases in all phases from development to response analysis. In addition, the respondent percentage yielded a small sample size making generalizable knowledge difficult. To note, the response rate is comparable to a national study assessing physician response rates [27].

## Conclusions

MSK education requires a multifaceted and multispecialty approach that can have several barriers to implementation. EM residency programs utilize a large variety of educational methods to transfer MSK knowledge. Although efforts are being made to address the known deficiency in MSK knowledge, further research is needed to perform a larger needs assessment, study innovative MSK education modalities, and develop a standardized MSK assessment for EM residency training.
